# Comparison of ^18^F-sodium fluoride PET/CT, ^18^F-fluorocholine PET/CT and diffusion-weighted MRI for the detection of bone metastases in recurrent prostate cancer: a cost-effectiveness analysis in France

**DOI:** 10.1186/s12880-020-00425-y

**Published:** 2020-03-02

**Authors:** Mathieu Gauthé, Kevin Zarca, Cyrielle Aveline, Frédéric Lecouvet, Sona Balogova, Olivier Cussenot, Jean-Noël Talbot, Isabelle Durand-Zaleski

**Affiliations:** 1 Nuclear Medicine, Hôpital Tenon, AP-HP, Sorbonne Université, 4 rue de la Chine, 75020 Paris, France; 2 AP-HP Health Economics Research Unit, INSERM UMR 1153, Paris, France; 3 Nuclear Medicine, Faculty of Medicine, Comenius University and St. Elisabeth Oncology Institute, Bratislava, Slovakia; 4 Radiology, Institut de Recherche Expérimentale Et Clinique, Cliniques Universitaires Saint-Luc, Université Catholique de Louvain, Brussels, Belgium; 5 Urology, Hôpital Tenon, AP-HP, Sorbonne Université, Paris, France; 6 Department of Public Health, Hôpital Henri Mondor, AP-HP,Université Paris 12, Créteil, France

**Keywords:** Prostate cancer, Medico-economic, Bone metastases

## Abstract

**Background:**

The diagnostic performance of ^18^F-sodium fluoride positron emission tomography/computed tomography (PET/CT) (NaF), ^18^F-fluorocholine PET/CT (FCH) and diffusion-weighted whole-body magnetic resonance imaging (DW-MRI) in detecting bone metastases in prostate cancer (PCa) patients with first biochemical recurrence (BCR) has already been published, but their cost-effectiveness in this indication have never been compared.

**Methods:**

We performed trial-based and model-based economic evaluations. In the trial, PCa patients with first BCR after previous definitive treatment were prospectively included. Imaging readings were performed both on-site by local specialists and centrally by experts. The economic evaluation extrapolated the diagnostic performances of the imaging techniques using a combination of a decision tree and Markov model based on the natural history of PCa. The health states were non-metastatic and metastatic BCR, non-metastatic and metastatic castration-resistant prostate cancer and death. The state-transition probabilities and utilities associated with each health state were derived from the literature. Real costs were extracted from the National Cost Study of hospital costs and the social health insurance cost schedule.

**Results:**

There was no significant difference in diagnostic performance among the 3 imaging modalities in detecting bone metastases. FCH was the most cost-effective imaging modality above a threshold incremental cost-effectiveness ratio of 3000€/QALY when imaging was interpreted by local specialists and 9000€/QALY when imaging was interpreted by experts.

**Conclusions:**

FCH had a better incremental effect on QALY, independent of imaging reading and should be preferred for detecting bone metastases in patients with biochemical recurrence of prostate cancer.

**Trial registration:**

NCT01501630. Registered 29 December 2011.

## Background

Prostate cancer (PCa) is the second most prevalent cancer in men worldwide, accounting for approximately 15% of all diagnosed cancers [1], presenting an annual incidence of 31.1 per 100,000 cases and being the 3rd cause of death by cancer in men behind lung and colorectal cancers [1].

Bone is the most frequent metastatic site of PCa and is the only metastatic site in approximately 62% of cases [2]. The prevalence of bone metastasis in PCa is 3% at diagnosis (for all stages), and its estimated cumulative incidence is 16.6% 5 years after PCa diagnosis [3]. The discovery of bone metastases marks a turning point in the history of the disease, especially in terms of treatment strategy and prognosis, and patients with bone metastases have a worse prognosis [3–5].

^18^F-sodium fluoride positron emission tomography/computed tomography (PET/CT) (NaF), ^18^F-fluorocholine PET/CT (FCH) and diffusion-weighted whole-body magnetic resonance imaging (DW-MRI) are three novel imaging techniques that are effective for the detection of PCa bone metastases and their results may impact the management of patients [6]. Their diagnostic performances in detecting bone metastases in PCa patients with first biochemical recurrence (BCR) have already been published [7–9]. However, to the best of our knowledge, their cost-effectiveness in this indication has never been compared.

The objective of this study was to provide evidence for policy making at the national level by comparing the cost-effectiveness of the detection of bone metastasis in PCa patients with first BCR by means of these three imaging modalities.

## Methods

The French multicentre study “FLUPROSTIC” (NCT01501630) was a prospective integrated clinical and economic national multicentre study comparing NaF, FCH and DW-MRI in detecting bone metastasis in PCa patients with first BCR. The medical objective was to compare the diagnostic performance and the impact on patient management of these three imaging modalities in this indication. It was completed by a cost effectiveness analysis using real treatment costs of trial patients. The FLUPROSTIC trial was conducted in accordance with the Declaration of Helsinki and approved by a national review board (IDRCB 2011-A01041–40). All patients provided written informed consent. The study design, inclusion/exclusion criteria, follow-up and standard of truth (SOT) for bone metastasis that were applied in this study are summarized in Additional file 1. The imaging protocols and imaging interpretation are detailed below. For the purpose of economic evaluation, we extrapolated the results of the trial using a state-transition model.

### Population

All PCa patients included in the FLUPROSTIC trial presenting with first BCR after previous definitive treatment for localized PCa, without ongoing androgen-deprivation therapy (ADT), were considered in this cost-effectiveness analysis. BCR was diagnosed after surgery, radiotherapy or alternative local treatment options with curative intent according to current recommendations [10]: two consecutive rising PSA values above 0.2 ng/ml following radical prostatectomy or any PSA increase greater than or equal to 2 ng/ml higher than the PSA nadir value, regardless of the nadir value, for non-surgical first-line definitive treatments (radiation therapy, brachytherapy, high-intensity focused ultrasound). Each patient was treated and followed up by his referring physician after the imaging workup according to standards of care (French Association Urology guidelines, which are similar to those of the European Association of Urology) [11, 12]. Patients for whom the SOT for bone metastasis was not feasible were excluded from the cost-effectiveness analysis because it was not possible to categorize them into a health state. Patients for whom the follow-up duration after the imaging workup was less than 1 year were also excluded from the medico-economic analysis, because it was not possible to evaluate their annual treatment costs.

### Imaging analysis

The imaging protocols are detailed in Additional file 2.

#### Pet/CT

A local nuclear physician with more than 5 years of experience in PET/CT reading prospectively read the PET/CT examinations the same day as the acquisition, not blinded to the results of other imaging, and provided a report of his analysis to the local clinicians (on-site reading of imaging). Two board-certified nuclear medicine physicians with 10 years of experience in PET/CT reading (one for NaF and one for FCH), blinded to the clinical and other imaging results, performed retrospective randomized readings independently of each other (central masked reading of imaging). All PET/CT examinations were reviewed in a three-panel mode, displaying CT-scan, FCH/NaF-scan and fusion images, using a dedicated workstation (Syngo.via, Siemens Healthcare).

For image quotation, the skeleton was parted into 8 regions: skull, thoracic cage, cervical, thoracic spine, lumbar spine, pelvis, humeri, and femurs. Any focal FCH or NaF uptake above the background in the bone not corresponding to a benign pathology on CT (around the joints, osteophytes, fractures, etc.) was reported as positive [9, 13].

#### MRI

A local oncoradiologist with more than 10 years of experience in MRI reading prospectively read the MRI examinations the same day as the acquisition, not blinded to the results of other imaging, and provided a report of his analysis to the local clinicians (on-site reading of imaging). A board-certified radiologist with more than 20 years of experience in bone marrow MRI imaging and cancer imaging, blinded to the clinical and other imaging results, performed retrospective randomized readings independently of the PET/CTs (central masked reading of imaging). All images were read on PACS workstations (Carestream Vue; Carestream Health).

The 8 anatomic regions of the skeleton were the same as those for PET/CTs. A focal bone metastasis was defined as a rounded focus larger than 5 mm with low signal intensity on T1-weighted images and for the evaluation of the whole whole-body MRI examination, of low signal intensity on T1, intermediate to high signal intensity on STIR, and high signal intensity on the high b-value DWI sequence. Diffuse bone metastasis was defined as low signal intensity of the bone marrow (lower than the signal intensity of disks and muscles) on T1, intermediate to high signal intensity on STIR, and high signal intensity on the high b-value DWI sequence [14, 15].

### Economic evaluation

The economic evaluation was conducted from the perspective of the French health care system. The short follow-up duration in the trial did not fully capture the medical and economic consequences of choosing between imaging techniques.

#### Model structure

**Fig. 1:**
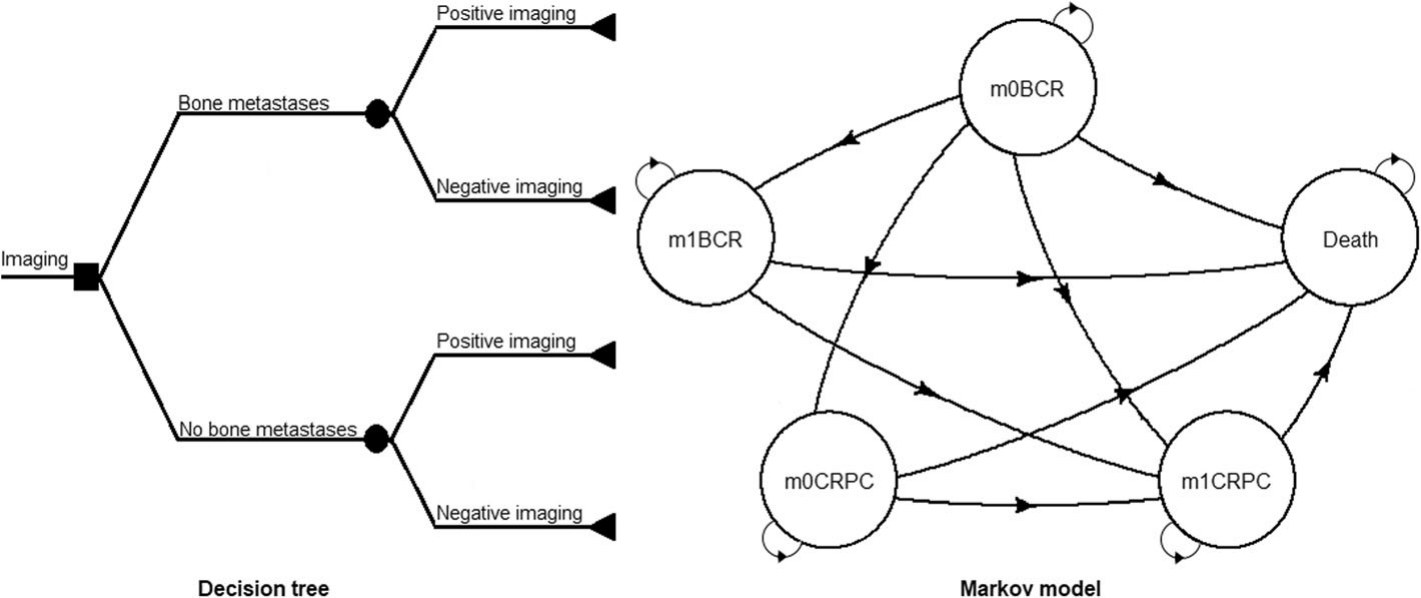
Decision tree combined with the Markov model used for evaluating costs and health-related outcomes. BCR = patients with first biochemical recurrence of prostate cancer; CRPC: patient with castration-resistant prostate cancer; m0 and m1: patient with and without bone metastases, respectively

We constructed a combination of a decision tree and Markov model based on the natural history of PCa (Fig. 1).

The decision tree was established by using the diagnostic accuracies of NaF, FCH and DW-MRI according to the established SOT for bone metastases. Markov models can be used to extrapolate the outcomes of a clinical trial over a longer time period, particularly in diseases with events that have ongoing risk or acute events that may occur more than once over the lifetime of a patient. In a Markov model, the course of a disease is represented by “states”: some chronic and some acute depending on the disease. The patient is always in one of a finite number of states of health referred to as Markov states. Everything that is important in the course of a disease is listed and the patient at each point in time has a given probability to either remain in that state or to transition to another. The probabilities of transitioning or not transitioning are calculated by increments of time, referred to as Markov cycles. During each cycle, the patient may make a transition from one state to another but not more, so the maximum duration of a cycle is medically defined. Moreover, based on the medical aspects of a disease, some transitions are possible, and others are not, e.g., the transition from “progression free” to “progressed” is possible, but the opposite is not. The probability of making a transition from one state to another during a single cycle is called a transition probability. The Markov model is defined by the probability distribution among the states and the probabilities of the transitions allowed. The “death” state is the absorbing state. For the purpose of economic analyses, each state is characterized by a cost and a weight that reflects the quality of life of a patient in that state [16].

Two analyses were thus conducted: one using the on-site reading accuracies of imaging and a second using the masked reading accuracies of imaging. To model the long-term health economics outcomes, we simulated a cohort of 10,000 PCa patients with first BCR at presentation identical to the trial patients, starting at the age of 70 years, which was the median age of the patients with first BCR in the FLUPROSTIC cohort, over a lifetime horizon. The Markov model had one-year cycles and consisted of 5 states: non-metastatic to bone (m0) BCR, metastatic to bone (m1) BCR, m0 castration-resistant prostate cancer (CRPC), m1-CRPC and death (Fig. 1). Patients with a correct diagnosis of bone metastases (true positives and true negatives of imaging) started in the m0-BCR or m1-BCR states depending on their bone metastatic status. Misdiagnosed nonmetastatic to bone patients (false positives of imaging) started in the m0-BCR state with the costs and quality of life of m1-BCR state for one cycle only. Misdiagnosed metastatic to bone patients (false negatives of imaging) entered the model in a tunnel state of one-cycle duration, and the diagnosis was corrected after 1 cycle as we noted in our series that the diagnosis of bone metastases was corrected on average during this time frame. Then, those patients entered m1-BCR, m1-CRPC or death states.

The state-transition probabilities associated with each health state were derived from the literature (Table 1) [2, 17–24]. A time-dependency was implemented for age, meaning that patients were aged 1 year each cycle.

**Table 1:** Health state annual transition probabilities and utilities used in the model

	**Transition probabilities (95% Confidence Interval)**	**References**
m0 BCR		
➔m1 BCR	0.0288 (0.0279–0.0297)	Hernandez 2018 [17]
➔m0 CRPC	0.0279 (0.0249–0.0308)	Hirst 2012 [18]
➔m1 CRPC	Combination P(m1 BCR + m0 CRPC)	
➔m0 BCR Death	French male mortality in 2017 (time-dependant)	Ined.fr [19]
On m0 BCR	1 - others P_m0 BCR_	
m0 CRPC		
➔m1 CRPC	0.1520 (0.1080–0.1940)	Smith 2005 [20]
➔m0 CRPC Death	Combination P(0.0413 + French male mortality in 2017)	Hirst 2012, Ined.fr [18, 19]
On m0 CRPC	1 – others P_m0 CRPC_	
m1 BCR		
➔m1 CRPC	0.2055 (0.1813–0.2251)	James 2015 [2]
➔m1 BCR Death	Combination P(0.1306 + French male mortality in 2017)	James 2015, Ined.fr [2, 20]
On m1 BCR	1 – others P_m1 BCR_	
m1 CRPC		
➔m1 CRPC Death	Combination P(0.2933 + French male mortality in 2017)	Fizazi 2011 [21]
On m1 CRPC	1 – others P_m1 CRPC_	
	**Utilities (Standard Deviation)**	**References**
m0 BCR	0.89 (0.14)	Torvinen 2013 [22]
m0 CRPC	0.86 (0.17)	Saad 2018 [23]
m1 BCR	0.74 (0.27)	Torvinen 2013 [22]
m1 CRPC	0.83 (0.13)	Lloyd 2015 [24]
Death	0	

*BCR* biochemical recurrence; *CRPC* castration-resistant prostate cancer; *m0* patient not metastatic to bone; *m1* patient metastatic to bone

#### Costs

Real treatment costs (updated to 2016 Euros (€)) were calculated for each patient included in the trial for the year following his inclusion. The costs of inpatients and outpatients were established based on activity logs completed by hospital practitioners, by considering the diagnosis-related group, healthcare common procedure coding system and ambulatory payment classification codes. The costs of out-of-hospital care (biology, imaging and medical consultations) were based on activity logs completed by the referring physician. Inpatients, outpatients and ambulatory care costs were calculated according to the data of the French National Study of health costs [25]. The costs of pharmaceuticals were based on prices listed by the French Public Welfare Agency [26]. The treatment costs that were used are summarized in Table 2. The detailed costs are provided in Additional file 3. We assumed that salvage radiation therapy of the prostatic lodge/pelvis was only performed once during the first year in eligible patients. Thus, its cost was considered only in the first cycle.

**Table 2:** Annual costs in Euros for non-metastatic and metastatic to bone prostate cancer patients with biochemical recurrence

Cost item	Patients without bone metastases (*n* = 48)	Patients with bone metastases (*n* = 7)
Androgen deprivation therapy	523 (364–695)	691 (244–1165)
Hospitalisation costs		
First year (with salvage radiation therapy)	2601 (1376–4381)	3706 (491–8138)
Other years	921 (431–1510)	3706 (631–7973)
Total costs		
First year	3524 (2277–5246)	4816 (1615–9234)
Subsequent years	1844 (1354–2434)	4815 (1689–9308)

Mean annual treatment/management costs with 95% confidence interval. Total costs included hospitalization costs, monitoring costs (office visit, biology and imaging) and drugs costs

The annual management costs of m0 and m1-CRPC patients were calculated in the same way based on a series of m0 and m1-CRPC patients who were included in the FLUPROSTIC trial. Thus, the mean total annual management costs for CRPC patients which were used to run our model were 5717€ (95% CI: 1634–11,869) for m0-CRPC patients and 12,346€ (95%CI: 3109–27,740) for m1-CRPC patients, based on a series of 15 patients including 4 patients with metastases. Misdiagnosed m0-BCR patients (false positive results of imaging) were attributed the costs of m0-BCR patients without counting the first-year salvage radiation therapy, and misdiagnosed m1-BCR patients (false negative results of imaging) were attributed the costs of m0-BCR patients for 1 year.

The annual production costs of each imaging modality per scan performed in the study hospital were calculated by adding the cost of the working time of each member of the staff involved in the care, the micro-costing and the depreciation of the PET/CT or MRI device (based on the buying cost, time to depreciate, annual maintenance and number of scans per year). The general costs of the medical centre were not considered for the calculation of the production costs as we assumed that they are the same for each imaging modality when performed in the same centre. The detailed production costs of each imaging modality are presented in Additional file 4.

A discount rate of 4% per annum was applied according to the current French recommendation for medico-economic studies [27].

#### Utilities

The utilities associated with each health state were derived from the literature (Table 1) [2, 17–24]. Misdiagnosed m0-BCR patients (false positive results of imaging) were attributed the utility score of m1-BCR patients and misdiagnosed m1-BCR patients (false negative results of imaging) entered the model in a tunnel state of one-cycle duration with the same utility as patients in the m0-BCR state.

#### Sensitivity analyses

Deterministic sensitivity analysis (DSA) was performed to evaluate the uncertainties surrounding relevant parameters within plausible ranges of 95% confidence intervals or standard deviations (according to the type of distribution), including the transition probability of developing bone metastasis, QALYs and treatment costs for m0 and m1-BCR patients.

A probabilistic sensitivity analysis was conducted to assess the effects of all parameter uncertainties, with a total of 1000 iterations of a Monte Carlo simulation. We used a binomial distribution for the transition probabilities of developing bone metastasis or resistance to castration and for QALYs, and a gamma distribution for treatment costs.

#### Model validation

We checked the internal validity of the Markov model by calculating the life expectancy of patients and comparing the results to French data [19].

### Statistical analysis

The data were analysed using R software. The heemod package [28] was used to calculate the transition probabilities from annual rates, run the Markov model, calculate the incremental cost-effectiveness ratio (ICER) and perform deterministic and probabilistic sensitivity analyses. The patient-based diagnostic performances of the three imaging modalities were compared by using the Cochran Q test with McNemar chi-square as a post hoc test. The agreement between the on-site and central readings for bone metastases was assessed for the 3 imaging modalities using Cohen’s kappa coefficient.

The economic evaluation followed the CHEERS recommendations [29].

## Results

### Base case: population and performance of imaging modalities

Of the 59 patients with biochemical recurrence enrolled in the FLUPROSTIC trial, 4 were excluded because the SOT could not be determined. The data of 55 BCR patients prospectively included between December 2011 and August 2014 were thus analysed. At least one bone metastasis was found in 7/55 (12.7%) patients (Table 3). The mean duration of patient follow-up was 3 years (range: 1–7 years).

**Table 3:** Characteristics of included prostate cancer patients with biochemical recurrence

Parameter	All patients	Patients without bone metastases (m0BCR)	Patients with bone metastases (m1BCR)
n	55	48	7
Median age in years [range]			
At prostate cancer diagnosis	65 [46–78]	65 [46–78]	66 [55–76]
At first biochemical recurrence	71 [50–87]	71 [50–86]	72 [56–87]
Initial group according to d’Amico classification			
Low risk	7 (13%)	7	0
Intermediate risk	23 (42%)	20	3
High risk	19 (35%)	15	4
Unknown	6 (10%)	6	0
Initial Gleason score			
≤ 6	13 (24%)	13	0
7	30 (55%)	25	5
≥ 8	8 (15%)	8	0
Unknown	4 (6%)	2	2
Initial International Society of Urological Pathologists (ISUP) 2014 grade group			
1	13 (24%)	13	0
2	17 (30%)	13	4
3	10 (18%)	10	0
4	7 (13%)	7	0
5	1 (2%)	1	0
Unknown	7 (13%)	4	3
First line treatment			
Surgery (prostatectomy ± lymph node dissection)	29 (53%)	28	1
Definitive radiation therapy ± ADT	19 (35%)	14	5
Other local treatment options*	7 (12%)	6	1
Median time to biochemical recurrence in months [range]	89 [4–228]	92 [4–228]	87 [6–149]
Median PSA serum value at BCR imaging workup ng/ml [range]	4.7 [0.2–137]	4.1 [0.2–52]	16.5 [1.0–137]
Management of biochemical recurrence after imaging workup			
Salvage radiation therapy (prostatic lodge ± pelvic lymph nodes) ± ADT	8 (15%)	8	0
ADT	27 (49%)	22	5
Surveillance	9 (16%)	9	0
Other treatment option**	11 (20%)	9	2

*ADT* androgen deprivation therapy; *: 4 brachytherapy and 3 high-intensity focused ultrasound (HIFU); **: 1 pelvic lymph node dissection, 7 HIFU, 1 cryoablation, 1 radiation therapy of an isolated bone metastasis and 1 surgery of 2 lung metastasis

There was no significant difference in diagnostic performance among the 3 imaging modalities in detecting bone metastases. The performances of each imaging modality in detecting bone metastases in BCR patients for on-site and central readings and the agreement between the two readings are presented in Table 4.

**Table 4:** Performances of imaging in detecting bone metastases of prostate cancer patients with first biochemical recurrence (patient-base analysis)

	Se	Sp	PPV	NPV	Accuracy	κ
NaF PET/CT						
On-site	71% (5/7)	92% (44/48)	56% (5/9)	96% (44/46)	89% (49/55)	0.96
Central	86% (6/7)	94% (45/48)	67% (6/9)	98% (45/46)	93% (51/55)	
FCH PET/CT						
On-site	43% (3/7)	100% (48/48)	100% (3/3)	92% (48/52)	93% (51/55)	0.86
Central	57% (4/7)	98% (47/48)	80% (4/5)	94% (47/50)	93% (51/55)	
DW-MRI						
On-site	57% (4/7)	83% (40/48)	33% (4/12)	93% (40/43)	80% (40/55)	0.59
Central	43% (3/7)	94% (45/48)	60% (3/5)	90% (45/50)	87% (48/55)	

*Se* sensitivity, *Sp* specificity, *PPV* positive predictive value, *NPV* negative predictive value

*NaF*^18^F-sodium fluoride, *FCH*^18^F-fluorocholine, *DW-MR*I diffusion-weighted whole-body magnetic resonance imaging

*Κ* Cohen’s kappa coefficient

**Fig. 2:**
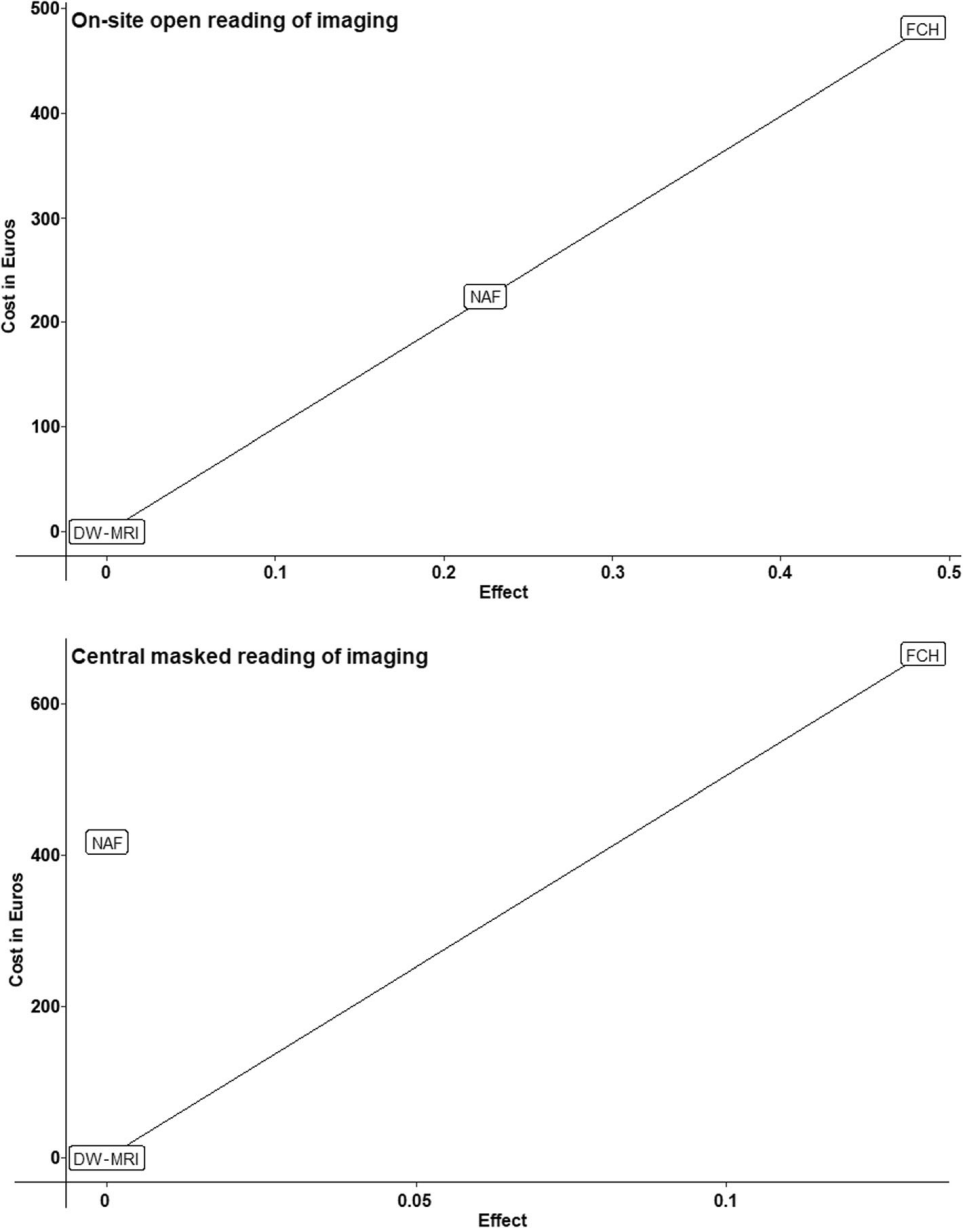
Incremental cost in Euros and effect of imaging strategies on the cost-effectiveness plane. NaF = ^18^F-sodium fluoride PET/CT; FCH = ^18^F-fluorocholine PET/CT; DW-MRI = diffusion-weighted whole-body magnetic resonance imaging

FCH was a more cost-effective imaging modality in both analyses. The economic evaluation using the on-site imaging reading accuracies showed an extended dominance of FCH compared to NaF (Table 5) (Fig. 2). The ICER was 993€ per QALY gained, with an incremental cost of 481€ and an average difference of 0.48 QALYs. For the economic evaluation using the central imaging reading accuracies, NaF was strictly dominated (Table 5) (Fig. 2). The ICER was 5055€ per QALY gained, with an incremental cost of 666€ and an average difference of 0.14 QALYs.

**Table 5:** Efficiency frontier and summary of cost-effectiveness results

**Efficiency frontier (on-site reading)**	**MRI with DW-MRI**	**NaF-PET/CT**	**FCH-PET/CT**
Life expectancy (years)	5.50	5.78	6.11
QALYs	4.45	4.67	4.93
Cost in Euros	22,160	22,385	22,641
ICER: Euros per QALY gained		1002	993
**Efficiency frontier (central reading)**	**NaF-PET/CT**	**MRI with DW-MRI**	**FCH-PET/CT**
Life expectancy (years)	5.87	5.87	6.03
QALYs	4.73	4.73	4.87
Cost in Euros	22,481	22,063	22,729
ICER: Euros per QALY gained	dominated		5055

*ICER* Incremental cost-effectiveness ration; *QALYs* Quality-adjusted life expectancy

### Sensitivity analyses

**Fig. 3:**
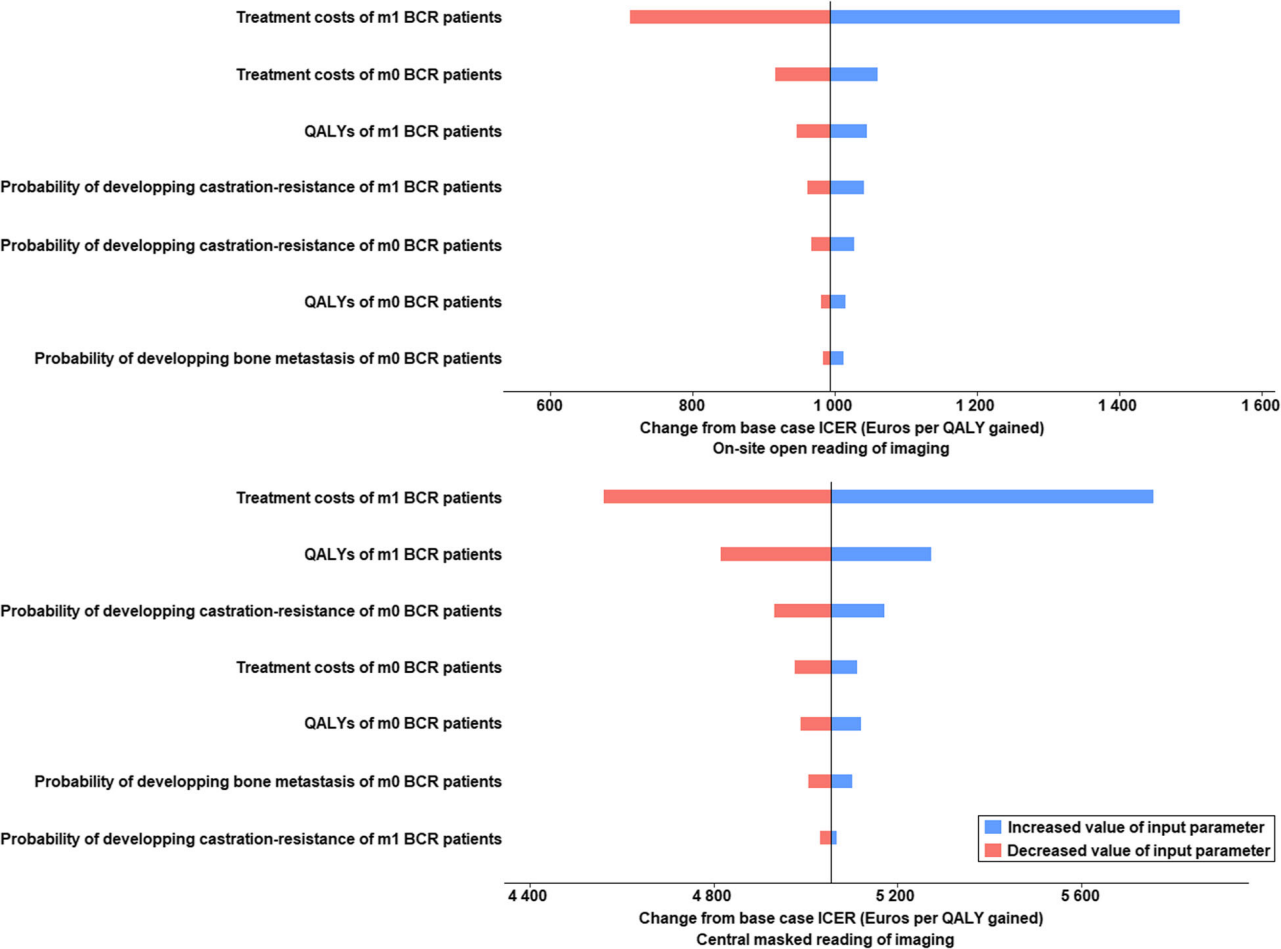
Tornado plot presenting uncertainties in costs in Euros within plausible ranges of the 95% confidence intervals. BCR = patients with first biochemical recurrence of prostate cancer; CRPC: patient with castration-resistant prostate cancer; m0 and m1: patient with and without bone metastases, respectively. The vertical line represents the base-case incremental cost-effectiveness ratio (ICER). QALY: quality-adjusted life year

The DSA showed that the uncertainty around the treatment costs of m1-BCR patients led in all cases to the largest variation in the ICER. These variations ranged from 730€ to 1547€ per QALY gained on the analyses using on-site imaging reading accuracies and from 4695€ to 5941€ per QALY gained on the analyses using central imaging reading accuracies (Fig. 3). All other tested parameters seemed to have minimal to moderate impact on the cost-effectiveness results (Fig. 3).

**Fig. 4:**
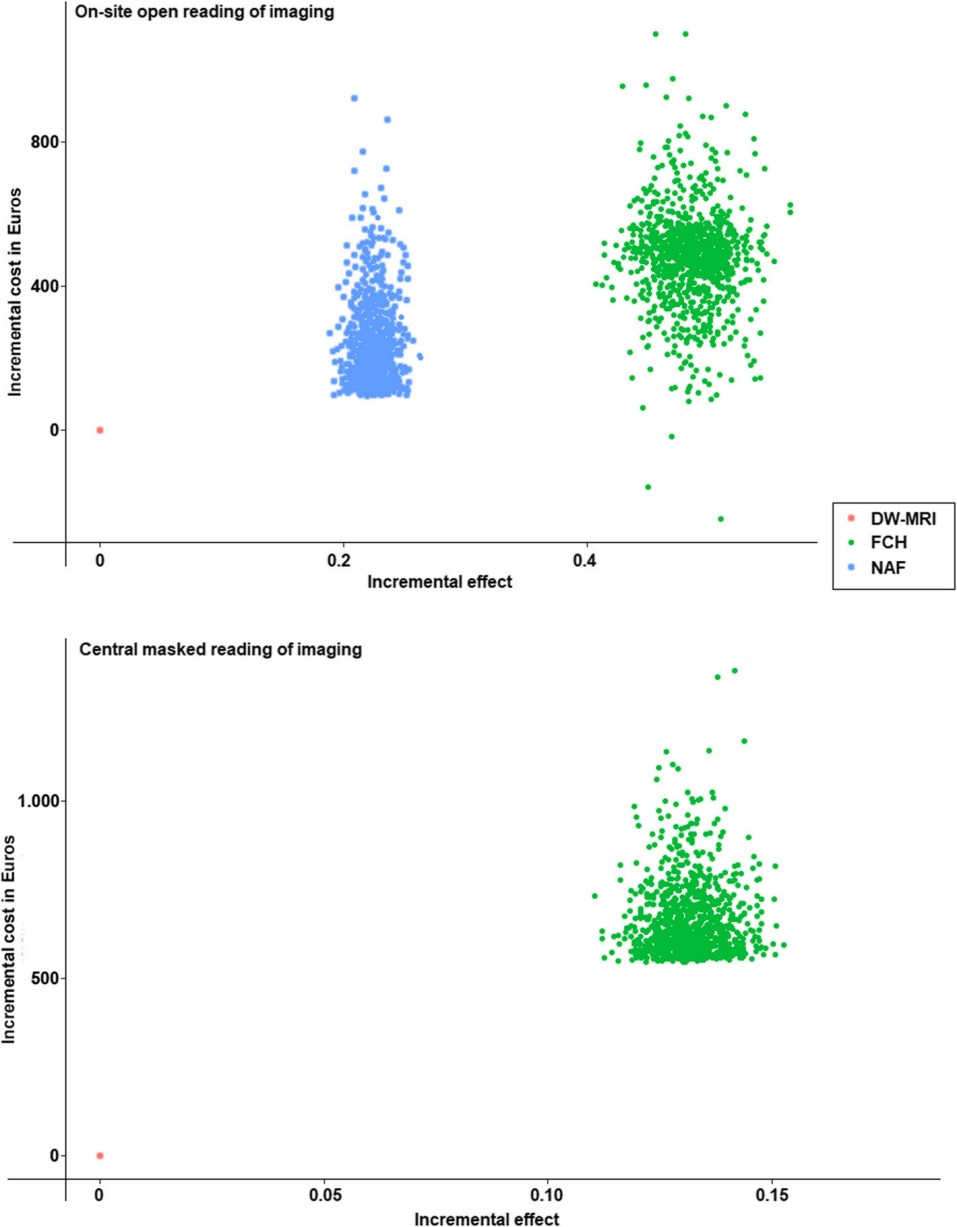
Scatter plot showing the uncertainty of the incremental cost-effectiveness ratio in Euros for each imaging modality. ICER = incremental cost-effectiveness ratio; NaF = ^18^F-sodium fluoride PET/CT; FCH = ^18^F-fluorocholine PET/CT; DW-MRI = diffusion-weighted whole-body magnetic resonance imaging

**Fig. 5:**
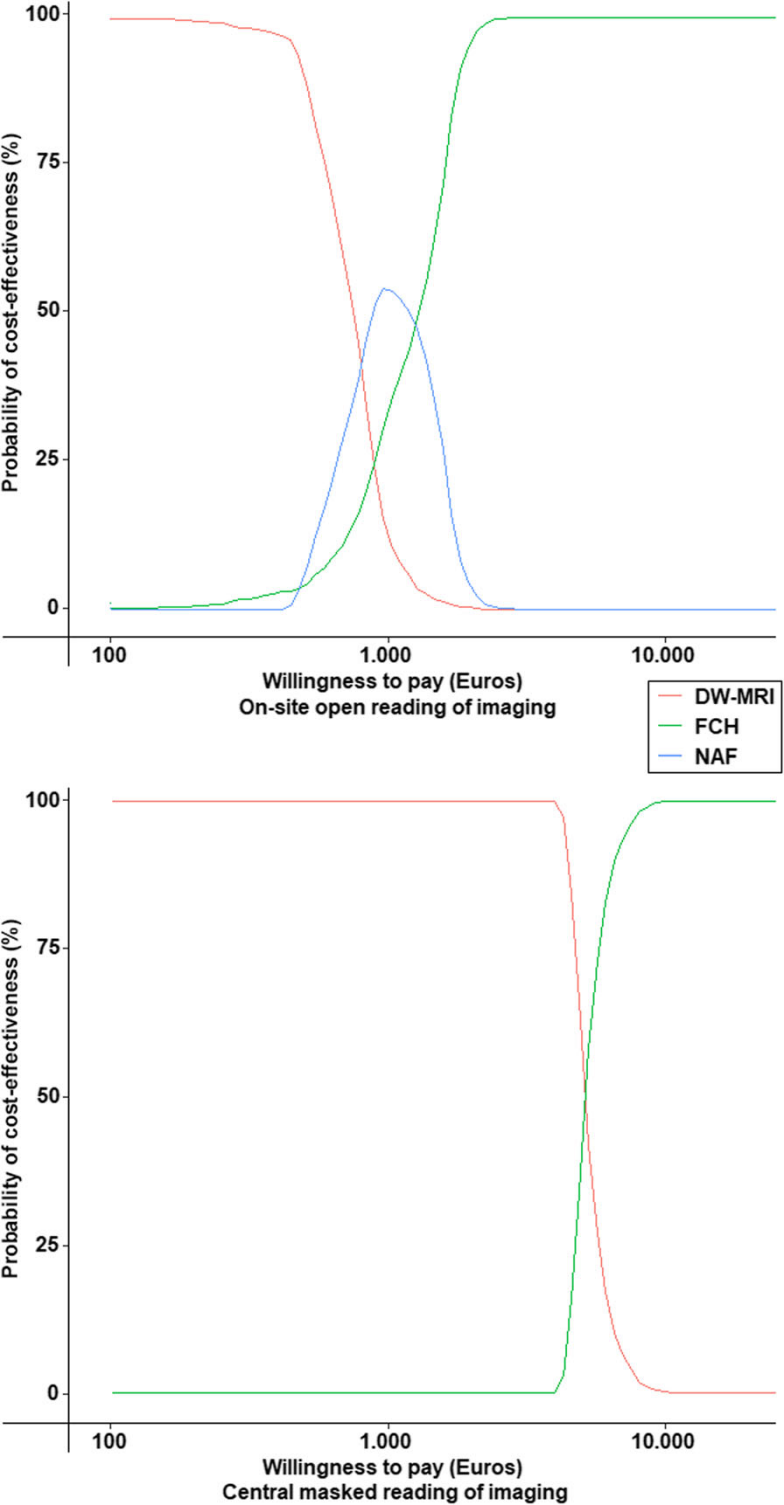
Cost-effectiveness acceptability curves in Euros showing the results of probabilistic sensitivity analyses for each imaging modality. NaF = ^18^F-sodium fluoride PET/CT; FCH = ^18^F-fluorocholine PET/CT; DW-MRI = diffusion-weighted whole-body magnetic resonance imaging

The probabilistic sensitivity analysis confirmed that FCH was the most cost-effective imaging modality in both analyses. It also confirmed the extended dominance of FCH compared to NaF when using on-site reading accuracies of imaging and that NaF was dominated when using the central imaging reading accuracies. The average incremental cost was 480€, the average difference was 0.47 QALYs and the average ICER was 1028€ for on-site imaging reading accuracy analyses and 649€, 0.14 and 5092€ respectively for central imaging reading accuracy analyses (Figs. 4 and 5). As shown in Fig. 5, FCH is probably the most cost-effective above a threshold ICER of 3000€ when imaging is interpreted by local specialists and of 9000€ when imaging is interpreted by experts.

We estimated that the overall life expectancy of PCa patients with BCR at the age of 70 years was 6.7 years, ranging from 4.1 years for m1-BCR to 9.2 years for m0-BCR, when running the model without considering imaging accuracies.

## Discussion

To the best of our knowledge, no previous study has ever reported a direct prospective head-to-head cost-effectiveness comparison of NaF, FCH and DW-MRI in the detection of bone spread in a homogenous group of PCa patients with first BCR.

### Imaging performances

The diagnostic performances of NaF, FCH and DW-MRI in this setting have already been published [7, 9, 30, 31]. The accuracy of NaF in detecting bone metastases was found to be significantly better than that of DW-MRI in a recent prospective comparison of 68 PCa patients with BCR [7]. Langsteger et al. observed a better lesion-based specificity of FCH compared to that of NaF (96% vs 91%, *p* = 0.033) for an equivalent sensitivity of 89% in the context of BCR [9]. In 2014, a meta-analysis compared the pooled performances of MRI (all variants, including DW-MRI) and choline PET/CT (pooling results of FCH and ^11^C-choline) in the diagnosis of bone metastases in PCa patients [30]. Overall, a non-significantly lower sensitivity was found for choline PET/CT compared to MRI, with pooled sensitivities of 87 and 95%, and without a difference in specificity values of 97 and 96% respectively. In 2016, Barchetti et al. performed FCH and DW-MRI in 152 PCa patients with BCR [31]. They considered the FCH results as the SOT. DW-MRI had a detection rate of 99% in patients presenting with bone spread on FCH [31]. In the present study, we found that the diagnostic performances of the 3 imaging modalities in detecting bone metastases in PCa patients with BCR were concordant with the reported values of other published studies. Thus, we assumed that our procedures did not favour any imaging strategy in the medico-economic analysis.

### Medico-economic analysis

In our study, FCH was always the most cost-effective imaging modality for staging patients with BCR, considering either the on-site reading by local specialists or the central reading by the experts of each imaging modality. PET/CT is often criticized for its higher cost compared to other imaging modalities that can be prescribed to explore PCa. From the point of view of an imaging centre, production costs are higher for the PET/CTs than for MRI (302€ for NaF and 881€ for FCH versus 112€ for DW-MRI), but these differences are only because of the radiotracers’ costs. Of note, no contrast agent was used for MRI in our study, which decreased MRI production costs as gadolinium is frequently injected into patients in routine practice for complementary sequences to DW-MRI. From the point of view of the French healthcare system, the reimbursed amounts for the PET/CTs, which are currently the same regardless of the radiotracer, are also approximately four times higher than those for MRI (Additional file 3). However, as shown in Table 5 and illustrated in Fig. 4, imaging costs have nearly no impact on overall patient care costs, as a difference of only 481€ and 666€ exists between the cheapest strategy (DW-MRI) and the most expensive strategy (FCH) when using on-site and central imaging performances respectively. On the other hand, FCH had higher QALY (0.48 and 0.14 with on-site and central imaging performances respectively) than DW-MRI.

### Limitations

The main factor shared by all studies addressing metastatic bone spread is the lack of histological evidence for most metastases, which were mainly characterized on the basis of follow-up data. However, this limitation applied equally to the 3 imaging modalities and it is assumed that it would not have favoured one of them.

The second major limitation of this work was the relatively limited number of included patients due to logistical difficulties in prospectively completing the entire imaging workup in the early 2010s. However, our study was the largest homogenous prospective head-to-head cost-effectiveness comparison of these 3 imaging modalities ever reported. The use of a Markov model of the disease was thus essential to simulate the outcomes of PCa patients with BCR in a larger number of patients on a lifetime horizon.

In this study we did not find significant differences among the 3 imaging modalities by using an analysis that summarized the performance of each imaging modality in detecting bone marrow involvement in a per-patient approach. The results could have been different by using a lesion-based analysis. However, we assume that this per-patient approach did not alter the impact on determining patient management or treatment costs, as in the real world therapeutic decision-making is based on a holistic approach of the patients’ disease (patient-based) and not on a lesion per lesion approach (lesion-based).

We found better interobserver agreement between on-site and masked readings for the PET/CTs than for DW-MRI. The low reproducibility of the DW-MRI readings may be explained by a lack of standardization of the analysis of this modality, which was recently prompted [32]. Indeed, the definition of bone, node and visceral metastases was settled by the time the study protocol was tailored, based on the available literature at that time, well before recent efforts for harmonization in image acquisition and reporting (MET-RADS criteria) [32]. Again, these differences in the reading agreement of imaging between on-site local specialists and central experts had almost no impact on the total cost of patient care.

The transition probabilities were extracted from the literature and are sometimes based on 10-year-old studies, whereas new therapeutics, such as second-generation anti-androgens such abiraterone acetate or enzalutamide, are now routinely prescribed to patients and may increase survival. Our model predicted an overall life expectancy of 6.7 years for PCa patients with BCR at the age of 70 years when running the simulation without considering the performance of imaging. This result was consistent with the average life expectancy of the French population of 79.4 for men in 2018 [19] and validated the model.

This study included patients with first BCR after previous definitive treatment for localized PCa. Some of them were treated with salvage high intensity focalized ultrasound (HIFU) after the imaging workup, through this alternative was not recommended at the time of patient inclusion in the FLUPROSTIC study. However, as HIFU is currently suggested in the EAU guidelines for the treatment of relapse for radiation-recurrent PCa [10], and as we used real treatment costs for the cost-effectiveness study, we assume our results may be extended to the current context of recurrent PCa. Furthermore, as the health-state costs are the same for the 3 imaging modalities, none were penalized by this point.

We chose to evaluate the medico-economic impact of imaging by considering only their performance in detecting bone metastases, while PCa patient management may be based on the detection of lymph node metastases, especially for oligometastatic patients. Performing a medico-economic analysis regarding the performance of imaging in detecting lymph node metastases would have required the availability of relevant transition probabilities in the literature in this setting which is currently lacking for such data. Such analysis could not have been performed for NaF, which only explores bone metabolism.

Finally, with the advent of PET/CT using ligands of prostate-specific membrane antigen (PSMA) as a radiotracer, the usefulness of such cost-effectiveness highlighting a metabolic radiotracer such as FCH for PCa might be questioned. However, we demonstrated that the imaging modality that was used did not impact the total cost of patient care but influenced QALYs (Fig. 4). Thus, the model we developed could be used to compare the cost-effectiveness of different imaging modalities, including PSMA ligands radiotracers.

## Conclusion

NaF, FCH and DW-MRI showed high diagnostic performances in detecting bone spread in prostate cancer patients with biochemical recurrence.

The cost-effectiveness analyses showed that imaging had no impact on the total costs of patient care.

FCH had a better incremental effect on QALY, independent of imaging reading, and should be preferred for imaging the biochemical recurrence of prostate cancer.

## Supplementary information

**Additional file 1.** FLUPROSTIC study design.

**Additional file 2.** Imaging protocols.

**Additional file 3.** Detailed treatment costs used in the model in Euros.

**Additional file 4.** Detailed real production costs in Euros per imaging modality.

## Data Availability

The datasets used and analysed during the current study are available from the corresponding author on reasonable request.

## Abbreviations

ADT: Androgen-deprivation therapy
BCR: Biochemical recurrence
CRPC: Castration-resistant prostate cancer
DSA: Deterministic sensitivity analysis
DW-MRI: Diffusion-weighted whole-body magnetic resonance imaging
FCH: ^18^F-fluorocholine PET/CT
ICER: Incremental cost-effectiveness ratio
M0: Non-metastatic to bone
M1: Metastatic to bone
NaF: ^18^F-sodium fluoride
PCa: Prostate cancer
PET/CT: Positron emission tomography / computed tomography
PSA: Prostate-specific antigen
QALY: Quality-adjusted life-year
SOT: Standard of truth
