# Supplementary material for: Comparison of 18F-sodium fluoride PET/CT, 18F-fluorocholine PET/CT and diffusion-weighted MRI for the detection of bone metastases in recurrent prostate cancer: a cost-effectiveness analysis in France
Source: BMC Med Imaging. 2020 Mar 2;20:25. doi: 10.1186/s12880-020-00425-y (PMC7052960; doi:10.1186/s12880-020-00425-y)
Supplement: Supplementary file 1 — Additional file 1. FLUPROSTIC study design. [file 12880_2020_425_MOESM1_ESM.docx]

Additional file 1: FLUPROSTIC study design

**Infrastructure and objectives**

The French multicentre FLUPROSTIC study was carried out through the « Support to Innovative and Costly Therapies » programme and promoted by the “Assistance Publique – Hôpitaux de Paris”. The main objective was to compare the diagnostic performance of ^18^F-sodium fluoride positron emission tomography/computed tomography (PET/CT), ^18^F-fluorocholine PET/CT and diffusion-weighted whole-body magnetic resonance imaging (DW-MRI) for the detection of first bone metastasis in prostate cancer (PCa) patients. Five French centres participated in patient recruitment. Imaging was performed in two centres that specialized in cancer imaging (Hôpital Tenon, Paris and ICO René Gauducheau, St-Herblain).

**Patient eligibility**

Nonmetastatic patients with PCa proven by biopsy were eligible. Inclusion was offered to any PCa patient suspicious for bone metastasis in any of the following settings: initial staging, biochemical recurrence without ongoing androgen-deprivation therapy (ADT), or castration-resistant prostate cancer. Patients were considered at risk of developing bone metastases if they presented any of the following situations : initial Gleason score 8–10, time to biochemical recurrence less than 3 years, initial stage T3b or higher, or a prostate-specific antigen (PSA) doubling time less than 3 months.

The exclusion criteria were other progressive neoplasias, therapy change during the imaging workup, imaging workup not completed within 1 month, and contraindications to any of the imaging modalities.

Upon providing informed consent, the patients were scheduled to undergo the 3 studied imaging modalities in a random order, in addition to the standard PCa recurrence workup.

**Follow-up**

Each patient was treated and followed-up by his referring physician after completing the imaging workup according to standards of care (French Association Urology guidelines which are comparable to those of the European Association of Urology).

The follow-up duration was at least 6 months after the imaging workup so that the standard of truth for imaging could be established. The follow-up duration for the medico economic analysis was at least 12 months to obtain real treatment costs.

**Standard of truth for bone metastases**

The presence of bone lesions was recorded in each bone region and summarized in a per-patient approach (presence or absence of bone marrow involvement). The presence of bone marrow involvement was determined during a panel review of all MRI and PET/CT examinations, based on the evaluation of all concurrent clinical, biological, histological and imaging data available, and of follow-up clinico-biological and imaging data obtained at least 6 months after the baseline examinations. The panel comprised one urologist, one radiation oncologist, one radiologist and one nuclear medicine physician, all with more than 10 years of clinical experience in PCa, belonging to Hôpital Tenon and who did not participate in the FLUPROSTIC study. The standard of truth (SOT) was constructed using the following steps for each patient. The panel reviewed MRI and PET/CT examinations, in combination with all concurrent and follow-up clinical, biological and imaging information. The reality of bone metastases (true positive of imaging) was first established based on the concordance of findings on imaging. For cases with at least one discordant reading, the panel reviewed all available material and adjudicated the examinations as being either positive or negative for bone marrow involvement in consensus, mostly by reading the PSA response (more than 50% compared to baseline value) to targeted PCa therapy (excluding ADT) and histological findings, if available.

The reality of the absence of bone metastases (true negative of imaging) was defined as an absence of bone metastasis at 6 months of follow-up according to all available material and adjudicated the examinations as being either positive or negative for bone marrow involvement in consensus.
