# Supplementary material for: Comparison of 18F-sodium fluoride PET/CT, 18F-fluorocholine PET/CT and diffusion-weighted MRI for the detection of bone metastases in recurrent prostate cancer: a cost-effectiveness analysis in France
Source: BMC Med Imaging. 2020 Mar 2;20:25. doi: 10.1186/s12880-020-00425-y (PMC7052960; doi:10.1186/s12880-020-00425-y)
Supplement: Supplementary file 2 — Additional file 2. Imaging protocols. [file 12880_2020_425_MOESM2_ESM.docx]

Additional file 2: Imaging protocols

PET/CT

Two PET/CT devices were used: GEMINI TF16 (Philips Medical Systems, Cleveland, Ohio, USA) in Hôpital Tenon and Biograph mCT (Siemens, Erlangen, Germany) in St-Herblain. For ^18^F-fluorocholine PET, dynamic images were acquired on the pelvis immediately after radiotracer injection of 2–3 MBq/kg of body mass (8 one-minute images); this step was followed by a “whole-body” acquisition from vertex to mid-thigh 20 minutes after FCH injection. For ^18^F-sodium fluoride PET, a whole-body acquisition from vertex to toes was performed 60 min after radiotracer injection of 3–4 MBq/kg of body mass. Low-dose CT without contrast-enhancement was performed prior to PET acquisition (120 kVp, 80 mA.s, slice thickness 2.5 mm, pitch 0.813, rotation time 0.5 s, FOV 600 mm) for attenuation correction and anatomic land-marking.

Duration of the PET images acquisition was 2 min per bed position.

MRI

Two MRI devices were used: 1.5 T Sonata MR (Siemens, Erlangen, Germany) in Hôpital Tenon and 1.5 T Ingenia (Philips, Best, The Netherlands) in St-Herblain. Patients were placed on the imaging table headfirst in the supine position and were covered with a head and neck coil, spine coils, and multi-element body matrix coils. Coronal 2D whole body T1- and STIR-weighted MR imaging pulse sequences, sagittal T1 and STIR sequences of the whole spine, and a transverse diffusion-weighted MR imaging (DWI) sequence were systematically performed. Detailed sequence parameters are presented in Additional file 2. For whole-boy MRI, five stacks of coronal T1 and STIR images, and axial DWI images were obtained, covering the body from the vertex to mid-thighs in 4 stacks. The whole spine was covered using three sagittal stacks. The DWI images were obtained using a Diffusion Weighted Imaging with Background signal Suppression (DWIBS) acquisition, based on a transversal inversion-recovery spin-echo echo-planar sequence (DWI IR SE-EPI) at 3 b values. The DWIBS images were reformatted in the coronal plane for side-by-side (stitched) analysis with the T1 and STIR images. The total acquisition time ranged between 38 and 45 minutes. Sequences were performed without contrast-enhancement. Additional optional sequences (transverse T2 TSE sequences for node screening) were not considered for the present study.

| Parameter | Body T1 | Body STIR | Spine T1 | Spine STIR | Body DWI |
| --- | --- | --- | --- | --- | --- |
| Plane | Coronal | Coronal | Sagittal | Sagittal | Transverse |
|  |  |  |  |  |  |
| Number of stations | 4 | 4 | 4 | 3 | 4 |
|  |  |  |  |  |  |
| Field of view [mm] | 300x500 | 300x500 | 400x275 | 400x275 | 300x500 |
|  |  |  |  |  |  |
| Matrix | 384x307 | 256x320 | 384x307 | 336x120 | 112x63 |
|  |  |  |  |  |  |
| Slice thickness [mm] | 5-6 | 5-6 | 4-5 | 4-5 | 5 |
|  |  |  |  |  |  |
| Slice gap [%] | 10 | 10 | 10 | 10 | 0 |
|  |  |  |  |  |  |
| Number of slices | 30 | 30 | 9-11 | 9-11 | 30 |
|  |  |  |  |  |  |
| Number of averages | 1 | 2 | 1 | 2 | 1 |
|  |  |  |  |  |  |
| TR [ms] | 550 | 3200 | 550 | 4358 | 3100 |
|  |  |  |  |  |  |
| TE [ms] | 15 | 50 | 15 | 64 | 63 |
|  |  |  |  |  |  |
| TSE factor | 4 | 15 | 4 | 30 | - |
|  |  |  |  |  |  |
| TI [ms] | - | 150 | - | 150 | 150 |
|  |  |  |  |  |  |
| Fat-suppression technique | - | STIR | - | STIR | STIR |
|  |  |  |  |  |  |
| b values [sec/mm²] | - | - | - | - | 0-500-1000 |

DWI= high b value diffusion-weighted imaging sequence; STIR= short tau inversion recovery sequence; T1= T1-weighted sequence; TE= echo time; TI= inversion time; TSE= turbo spin echo
