# Supplementary material for: Comparison of 18F-sodium fluoride PET/CT, 18F-fluorocholine PET/CT and diffusion-weighted MRI for the detection of bone metastases in recurrent prostate cancer: a cost-effectiveness analysis in France
Source: BMC Med Imaging. 2020 Mar 2;20:25. doi: 10.1186/s12880-020-00425-y (PMC7052960; doi:10.1186/s12880-020-00425-y)
Supplement: Supplementary file 3 — Additional file 3. Detailed treatment costs used in the model in Euros. [file 12880_2020_425_MOESM3_ESM.docx]

Additional file 3: Detailed treatment costs used in the model in Euros

| Cost item | Euros |
| --- | --- |
| Drug annual costs per patient |  |
| Abiraterone acetate | 36859 |
| Bicalutamide | 488 |
| Dutasteride | 232 |
| Estramustine | 1,504 |
| Gosorelin | 1,319 |
| Leuprolide acetate | 1,319 |
| Nilutamide | 1,627 |
| Triptorelin | 1,319 |
|  |  |
| Inpatients annual costs per diagnosis-related group |  |
| Respiratory neoplasme without CC/MCC | 1102 |
| Heart failure and shock with CC | 3877 |
| Medical back problems without MCC | 2215 |
| Medical back problem with MCC | 4391 |
| Transurethral prostatectomy without CC/MCC | 2399 |
| Transurethral prostatectomy with CC/MCC | 3293 |
| Major male pelvic procedure without CC/MCC | 8069 |
| Other male reproductive system or procedure for malignancy without CC/MCC | 2422 |
| Other male reproductive system or procedure excluding malignancy without CC/MCC | 613 |
| Myeloproliferative disorder or poorly differenciated neoplasma without CC/MCC | 6133 |
|  |  |
| Outpatient per session’s costs |  |
| Intensity modulated radiotherapy, per session | 165 |
| Stereotactic radiotherapy, per session | 947 |
| Chemotherapy | 1135 |
|  |  |
| Ambulatory care annual costs |  |
| Urologist | 115 |
| Prostate-specific antigen serum level assay | 75 |
| Multiparametric magnetic resonance imaging of the pelvis | 267 |
| PET/CT | 1090 |

CC: complication or comorbidity; MCC: major complication or comorbidity

Treatment costs (drugs, inpatients, outpatients and ambulatory care) in Euros, according available data of French Public Welfare Agency and French National Study of health costs for year 2016 (inpatients and outpatients) and 2017 (drugs and ambulatory care)
