# Supplementary material for: Comparison of 18F-sodium fluoride PET/CT, 18F-fluorocholine PET/CT and diffusion-weighted MRI for the detection of bone metastases in recurrent prostate cancer: a cost-effectiveness analysis in France
Source: BMC Med Imaging. 2020 Mar 2;20:25. doi: 10.1186/s12880-020-00425-y (PMC7052960; doi:10.1186/s12880-020-00425-y)
Supplement: Supplementary file 4 — Additional file 4. Detailed real production costs in Euros per imaging modality. [file 12880_2020_425_MOESM4_ESM.docx]

Additional file 4: Detailed real production costs in Euros per imaging modality

| Cost item | Euros |
| --- | --- |
| PET/CT (scans = 3115) |  |
| Staff | 60.0 ± 1.2 |
| Supplies | 3.5 ± 0.1 |
| Device depreciation | 75.4 ± 3.8 |
| Sodium ^18^F-fluoride (n=164) | 163.5 ± 8.2 |
| ^18^F-fluorocholine (n=852) | 742.8 ± 37.1 |
|  |  |
| MRI (scans =5039) |  |
| Staff | 64.8 ± 0.7 |
| Supplies | 0.4 ± 0.2 |
| Device depreciation | 46.6 ± 2.3 |
| Contrast agent | 0 (not used in the study) |

Mean production costs (± standard deviation) in Euros per imaging modality

n = number of scans
